# Efficacy of short-term splint immobilization in the treatment of pediatric discoid lateral meniscus after saucerization management

**DOI:** 10.1097/MD.0000000000033553

**Published:** 2023-04-14

**Authors:** Yao Liu, Ya Liu, Lun-Qing Zhu, Yun-Fang Zhen, Fu-Yong Zhang, Xiao-Dong Wang

**Affiliations:** a Department of Orthopaedics, Children’s Hospital of Soochow University, Suzhou, Jiangsu Province, China.

**Keywords:** meniscus, pediatric, physical therapy, rehabilitation, saucerization

## Abstract

There are no universal guidelines for rehabilitation after saucerization for children with discoid lateral meniscus. This study determined if short-term knee splint immobilization and delayed rehabilitation produces the same benefit as early rehabilitation after saucerization in children, in terms of knee function and pain intensity. A retrospective review was performed by categorizing patients into 2 groups depending on whether a splint immobilization was adopted postoperatively: for group A, rehabilitation began early without splint immobilization after surgery, and for group B, a knee splint was immobilized for 2 weeks. Numerical rating scale scores were collected in patients 1, 3, and 7 days, Lysholm scores were measured at 4 and 8 weeks postoperatively, and the gradual return to normal activities was documented. Forty-eight patients and 53 knees were included: group A had 30 patients with 31 knees, and group B had 18 patients with 22 knees. There was no improvement in numerical rating scale scores on the 1^st^ (*P=*.519), 3^rd^ (*P=*.421), and 7^th^ (*P=*.295) postoperative days in group B. The Lysholm scores of group A (62.94 ± 8.68) was higher than that of group B (46.68 ± 9.82) measured 4 weeks following surgery, but there was no difference at 8 weeks (*P=*.237), and both groups had similar time to return to normal activities (*P=*.363). For discoid lateral meniscus patients who underwent isolated saucerization, short-term splint immobilization did not significantly help relieve postoperative pain. There was a comparable time-course for return to normal activities in both study groups.

## 1. Introduction

Meniscal rim preservation through partial meniscectomy is currently recommended for a symptomatic discoid lateral meniscus (DLM) in children. ^[1,2]^ However, there is no consensus for type or extent of rehabilitation after surgery to promote return to normal function. For patients undergoing saucerization treatment of the lateral meniscus without splint immobilization, physical therapies starting 2 weeks after surgery is generally advised,^[3]^ but others recommend an early rehabilitation as soon as postoperative pain is tolerable.^[4]^ While much has been written about discoid menisci in adults, less is known about postoperative therapies in the pediatric population.

Surgery can be painful, and this may negatively impact the well-being of children after the procedure. Pain may adversely affect postoperative recovery including limiting postoperative joint movement and reducing muscle strength. Postoperative pain is most commonly treated with analgesics, but often there is insufficient relief.^[5]^ Meanwhile, most pediatric patients have weak self-control and tend to be hyperactive. Early aggressive activity after surgery can result in inflammation and chronic pain.^[6]^ Splints are widely used in pediatric patients. Basic indications of splinting include protecting operative sites, controlling pain, and decreasing bleeding or swelling.^[7]^ A previous study showed 2-week period immobilization had no statistical differences in range of motion (ROM) and thigh muscle strength after anterior cruciate ligament reconstruction when compared to 3-day period immobilization.^[8]^ However, excessive use of splint or brace after surgery can lead to joint stiffness and muscle weakness.^[9,10]^ Consequently, a short-term splint immobilization is applied in our institution for children with DLM after surgery.

The purpose of this retrospective study was to determine whether short-term splint immobilization would be of benefit to the patient by relieving postoperative pain, and to determine if the splint fixation would affect the time-course for postoperative recovery.

## 2. Methods

### 2.1. Study design (patients and methods)

Ethical approval for this retrospective study was obtained from the ethics committee of Children’s Hospital of Soochow university (2022CS170). All patients and their parents gave informed consent prior to participating in the study. This is a single-institution, retrospective review of archived data between 2015 to 2020 for patients undergoing treatment for DLM.

Initially, patients enrolled between 2015 to 2017, who received saucerization without repair of the meniscus were treated with early-stage rehabilitation. Immediate weight bearing without external immobilization was advised to begin on the first postoperative day, and continuous passive motion was initiated after pain relief. However, some patients had difficulty to adhere to medical advice to initiate early rehabilitation because of postoperative pain. After 2018, we chose another line of treatment, a short-term knee immobilizing splint fixation for 2 weeks, and then rehabilitation therapy was initiated. To correlate the short-term brace immobilization with the postoperative degree of pain and functional outcomes, patients were divided into 2 groups: group A was treated between 2015 and 2017, and group B was treated between 2018 and 2020.

Patient Inclusion criteria were the following: <18 years of age; Diagnosed with symptomatic discoid lateral meniscus; Underwent saucerization surgery in the absence of other treatment; Had adequate clinical documentation; Were able to be followed for more than 6 months. The exclusion criteria were as follows: Patients received saucerization with repair of the meniscus; Inadequate documentation; Patients received femoral nerve blockade during perioperative period; Insufficient follow-up.

The numerical rating scale (NRS) scores was used to assess postoperative pain in which 0 indicates no pain, and 10 severe pain.^[11]^ The Lysholm Knee Scoring Scale refers to instability and postoperative outcomes, which were used to evaluate clinical outcomes.^[12]^ After data collection age, gender, duration of symptoms, type of DLM according to Watanabe classification,^[13]^ body mass index (BMI), operation time, NRS at 1, 3, and 7 days postoperatively, Lysholm scores at 4 and 8 weeks, and time to return to normal activities were evaluated. Information was mined from inpatient and outpatient medical records, and telephone follow-up as required.

### 2.2. Surgical treatment

All patients with DLM received general anesthesia. Two specialists performed surgical interventions, routine diagnostic arthroscopic inspection of the knee joint was performed, and the presence of a discoid meniscus and the type of DLM was confirmed. Meniscus saucerization was executed by using meniscal baskets, meniscectomy and shavers, starting at the free edge to respect the central portion piece-by-piece. Saucerization was performed cautiously, proceeding until a 6 to 8 mm residual peripheral rim was achieved. The surgeon also searched for meniscus tears and assessed meniscal stability. When meniscus margin hypertrophy and internal horizontal tears were observed, the inferior or superior leaf was resected. Additional interventions such as all inside, inside-out or outside-in repairs were considered based on the types of meniscus tears or meniscus stability.^[1,3]^

### 2.3. Postoperative rehabilitation

Since postoperative hospitalization was 1 to 2 days, patients received home exercise program and information from physical therapist without supervision. In group A, starting on the day after surgery, partial weight bearing on the leg was advised with the use of crutches. Ice and compression therapy was used to reduce inflammation and swelling within 2 weeks. The patient underwent isometric contraction training of the quadriceps and hamstring muscles at an early-stage depending on pain tolerance levels. Passive ROM started at the same time, progressing to dynamic proprioceptive training. For those in group B, the knee immobilizing splint was placed after surgery. Then, protected weight bearing was initiated, and quadriceps active contraction and ankle pump training were used. Gradual progressive ROM was started after removing the splint at 2 weeks postoperatively.

### 2.4. Statistical analysis

IBM SPSS Statistics for Windows, version 25.0 (IBM Corp., Armonk, NY) was used for statistical analysis. Parametric and nonparametric tests assessed statistically significant differences between groups. Between-group differences in patients ages were compared using the student *t* test. The Mann–Whitney *U* test was used to compare differences in BMI, duration of symptom, surgical time, NRS scores at 1, 3, and 7 days postoperatively, Lysholm scores at 4 and 8 weeks, and time to return to sport. The Chi-squared test was used for between-group comparisons of gender distribution and affected side. *P* values < .05 were considered statistically significant.

## 3. Results

### 3.1. Patient enrollment and exclusion

For group A, 53 patients who suffered from DLM were treated between 2015 and 2017. In total, 18 patients were excluded because they underwent saucerization with repair of meniscus and 5 patients were excluded because of incomplete clinical data. There were 30 patients, 16 male and 14 female with 31 knees eligible for inclusion in the study. The average age at hospitalization was 10.8 years (range, 6 to 14.7 years). About 21 knees of discoid menisci were classified as Watanabe I and 10 knees were Watanabe Ⅱ. The mean preoperative duration of symptoms was 7.2 months (range, 0.25–24 months).

For group B, 60 patients with symptomatic DLM were treated at the hospital between 2018 and 2020. Overall, 34 patients who received saucerization plus meniscus fixation and 4 patients received femoral nerve blockade were excluded, while there was inadequate follow-up in 4 patients. Thus, 18 patients, 7 males, and 11 females with 22 knees were included. The average age was 10.1 years (range, 5.6–14.3 years). There were 14 cases with Watanabe type I discoid menisci and 8 cases with Watanabe type II. The mean preoperative duration of symptoms was 8.9 months (range, 0.3–24 months).

There were no significant differences in patient characteristics including age, sex distribution, BMI, type of DLM and duration of symptoms (see Table 1). Both groups had similar surgical time (*P=*.409).

**Table 1:** Comparison of patient characteristics between groups.

Parameters	Group A	Group B	*P* value
Mean (SD) age (yr)	10.8 (2.59)	10.2 (2.39)	.430
Sex (male/ female) *	16/15	8/14	.207
Mean (SD) body mass index	17.8 (1.93)	17 (1.11)	.942
Type of DLM according to Watanabe† (type I/type II)	21/10	14/8	.755
Mean symptoms duration (mo)	7.2	8.9	.792

DLM = discoid lateral meniscus, SD = standard deviation.

* There were 48 patients and 53 knees were included in the study (the early-stage group: 30 patients with 31 knees, the late-stage group: 18 patients with 22 knees).

† The Watanabe classification describes three types of discoid menisci: type I is a stable, complete discoid meniscus; type II is a stable, partial discoid meniscus; and type III is an unstable discoid meniscus with the lack of any posterior monoconidial attachments.^[13]^

### 3.2. Clinical data

There was no improvement in NRS pain scores on the 1st (*P=*.519), 3rd (*P=*.421), and 7th (*P=*.295) postoperative days in group B compared to group A (Table 2). The mean Lysholm score of group A was significantly higher than that of group B at 4 weeks follow-up (62.94 ± 8.68 vs 46.68 ± 9.82, *P<*.01). However, at 8 weeks postoperatively, there was no statistically significant difference (*P=*.237), and both groups returned to normal activities (*P=*.363) (Table 3) over a similar time-course.

**Table 2:** Comparison of self-reported numerical rating scale (NRS) pain scores* between groups.

Postoperative time (d)	Group A Mean NRS score (SD), (n=31)	Group B Mean NRS score (SD), (n=22)	*P* value
1	2.6 (0.97)	2.5 (0.72)	.519
3	1.1 (0.80)	0.9 (0.62)	.421
7	0.2 (0.49)	0.1 (0.29)	.295

SD = standard deviation.

* No pain=0; worst pain=10.

**Table 3:** Comparison of patient outcomes between groups.

Parameters	Group A	Group B	*P* value
Mean *Lysholm scores (SD) at 4 weeks postoperatively	62.9 (8.68)	46.7 (9.81)	<.001
Mean Lysholm scores (SD) at 8 wk postoperatively	91.88 (4.88)	89.3 (7.33)	.237
Mean time (wk) to return to normal exercise (SD)	9 (1.87)	9.5 (2.13)	.363

SD = standard deviation.

* Lysholm scores, out of 100 points, where 100 = best possible score and 0 = worst possible score.

## 4. Discussion

The DLM is a congenital abnormal meniscus variant with an incidence of 10% to 13% in Asia, 3% to 5% in the West. ^[3,14]^ Patients with DLM are prone to injury due to the unusual shape and size of the meniscus. Conservative approaches are adopted for the treatment of asymptomatic or minimal symptomatic DLM. Operation should be considered when the patient presents with persistent symptoms, including pain, blockage, edema, or limitation of normal activities. ^[15]^ The current study advocates restoring a stable and as near to normal meniscus, especially in younger patients.^[3]^

Arthroscopic saucerization is a type of partial meniscectomy, consisting of removal of the central portion of the meniscus, and restoring its “C” shape in order to preserve its function of absorbing and distributing weight loads.^[2,16]^ Despite increasing emphasis on meniscus repair, arthroscopic saucerization without repair of menisci still accounts for the largest proportion in the treatment methods of pediatric symptomatic DLM.^[6]^ However, there is no universal agreement regarding rehabilitation protocols that will be beneficial to patients, especially in children. For adults who have undergone isolated meniscus plasty, early postoperative rehabilitation has been advocated. However, children have poor compliance with functional exercises,^[17]^ and postoperative pain hinders rehabilitation despite the use of analgesics. Knee immobilizers are commonly used for postoperative pain management.^[18]^ Splints can decrease bleeding and protect operative sites.^[7]^ There is a dearth of information regarding the risk/benefit ratio of short-term knee immobilization with a splint following DLM after arthroscopic saucerization in children.

There are several prognostic factors for DLM reported in the literature. A retrospective study with 502 patients showed that 4 variables, including male sex, BMI < 18.5 kg/m2, age of onset < 14 years, and symptoms duration < 24 months are related to good postoperative outcomes.^[19]^ A previous study noted that obesity is associated with worsening knee function after arthroscopic partial meniscectomy,^[20]^ and Kose et al^[21]^, showed that the age at the time of surgery was the only predictor that correlated with the Lysholm score at the final follow-up. In our work, there was no statistically significant difference between age, gender, BMI and symptom durations, thus potentially reducing outcome bias.

Because of enhanced focus on meniscus stability and meniscus repair, after 2018, a smaller percentage of DLM patients received saucerization alone in group B. We acknowledge that some patients who should have received saucerization plus meniscus repair prior to 2018 only received saucerization for DLM, which may have affected the course and choice of postoperative rehabilitation. However, studies comparing saucerization alone and meniscus plasty with fixation noted only small differences in the degree of long-term knee degeneration and showed no significant clinical differences in short-term follow-up.^[22,23]^

Due to the short length of hospital stay after DLM operation, patients with DLM might not receive adequate pain control. Peripheral nerve blockade has been shown to improve postoperative analgesia while decreasing the requirement for opioids in the pediatric population.^[24]^ Considering the small number of cases using peripheral nerve block during the study period, they were not recorded and evaluated. Postoperative pain may cause adverse effects on surgical outcome, including limited ROM and resistance to rehabilitation. There have been increasing efforts to improve the perioperative pain management in children, but it is still suboptimal.^[25]^ Rest, ice, compression, elevation, bracing, and oral analgesics including acetaminophen, nonsteroidal anti-inflammatory drugs are common treatments for postoperative pain.^[18]^ Knee immobilizers and braces are commonly used both in children and adults after surgery. However, our study showed 2 weeks brace immobilization did not significantly improve postoperative pain control. A similar conclusion was documented by Hiemstra et al^[26]^, where 88 patients underwent hamstring tendon anterior cruciate ligament reconstruction. Postoperatively, patients were randomly assigned to either the immobilized group or the no immobilizer group, data showed that there was no difference in pain scale scores nor medication consumed at 2 to 3 weeks.

In terms of recovery time, our study indicated the Lysholm score of group A was significantly better than that of group B 4 weeks after surgery. However, there was no significant difference between the 2 groups in measurable knee function at 8 weeks postoperatively. Time to return to normal exercise after surgery did not differ significantly between the 2 groups. Short-term splint immobilization may delay the recovery of knee function in early-stage but has no significant effect on the final outcome. Goodwin and Morrissey reported that physical therapy had little or no effect on return to daily activities in adult patients with arthroscopic partial meniscectomy.^[27]^ Another study compared early versus delayed physical therapy, and showed that there was no significant difference in patients who received weight bearing at 3 or 10 days postoperatively.^[28]^

Our study does have limitations that need to be documented. Due to the retrospective nature of this study, the data on ROM or muscle strength were not complete. It was a retrospective study with low patient enrollment. Randomized clinical trials are needed to provide additional evidence-based findings. All the surgeries were performed by 2 attendings, the result of the study may not be generalized. The form of rehabilitation training in our study was home exercise, where patients were provided with an exercise program and information, but patients did not receive supervision from a rehabilitation therapist. Because of this, it is likely that the duration, frequency, and intensity of rehabilitation training was inadequate. However, a prospective randomized study by Jokl et al^[29]^ indicated no differences in subjective evaluation of knee function and the ability to resume work between outpatient physical therapy compared to home exercise following arthroscopic knee surgery. Additionally, a meta-analysis comparing outpatient physical therapy plus home exercise versus home exercise alone after arthroscopic partial meniscectomy, demonstrated a 9° increase in knee flexion ROM, but there were no significant clinical changes between groups.^[30]^

## 5. Conclusion

This study found that male and female pediatric DLM patients with arthroscopic saucerization alone, and short-term knee splint immobilization did not have reduced postoperative pain, and there was no effect on the time to return to normal activities.

## Author contributions

**Conceptualization:** Yao Liu.

**Data curation:** Yao Liu, Ya Liu, Lun-Qing Zhu, Yun-Fang Zhen.

**Formal analysis:** Yao Liu, Ya Liu, Lun-Qing Zhu, Xiao-Dong Wang.

**Investigation:** Ya Liu, Lun-Qing Zhu.

**Project administration:** Fu-Yong Zhang.

**Resources:** Yao Liu, Ya Liu.

**Supervision:** Ya Liu, Lun-Qing Zhu, Yun-Fang Zhen, Fu-Yong Zhang, Xiao-Dong Wang.

**Writing – original draft:** Yao Liu.

**Writing – review & editing:** Yao Liu, Ya Liu, Lun-Qing Zhu, Yun-Fang Zhen, Fu-Yong Zhang, Xiao-Dong Wang.
